# Care staff and family member perspectives on quality of life in people with very severe dementia in long-term care: a cross-sectional study

**DOI:** 10.1186/s12955-014-0175-3

**Published:** 2014-12-09

**Authors:** Linda Clare, Catherine Quinn, Zoe Hoare, Rhiannon Whitaker, Robert T Woods

**Affiliations:** School of Psychology, Bangor University, Bangor, Gwynedd LL57 2AS UK; NWORTH, Bangor University, Bangor, UK; DSDC Wales, Bangor University, Bangor, UK

**Keywords:** QUALID, Family caregivers, Staff attitudes, Well-being

## Abstract

**Background:**

Little is known about the quality of life of people with very severe dementia in long-term care settings, and more information is needed about the properties of quality of life measures aimed at this group. In this study we explored the profiles of quality of life generated through proxy ratings by care staff and family members using the Quality of Life in Late-stage Dementia (QUALID) scale, examined factors associated with these ratings, and further investigated the psychometric properties of the QUALID.

**Methods:**

Proxy ratings of quality of life using the QUALID were obtained for 105 residents with very severe dementia, categorised as meeting criteria for Functional Assessment Staging (FAST) stages 6 or 7, from members of care staff (n = 105) and family members (n = 73). A range of resident and staff factors were also assessed.

**Results:**

Care staff and family member ratings were similar but were associated with different factors. Care staff ratings were significantly predicted by resident mood and awareness/responsiveness. Family member ratings were significantly predicted by use of antipsychotic medication. Factor analysis of QUALID scores suggested a two-factor solution for both care staff ratings and family member ratings.

**Conclusions:**

The findings offer novel evidence about predictors of care staff proxy ratings of quality of life and demonstrate that commonly-assessed resident variables explain little of the variability in family members’ proxy ratings. The findings provide further information about the psychometric properties of the QUALID, and support the applicability of the QUALID as a means of examining quality of life in very severe dementia.

Little is known about the quality of life of people with very severe dementia [1], and research evidence in this area is lacking [2]. An important goal of long-term care provision for this group should be to promote quality of life [2], and in order to achieve this goal it is essential to understand as much as possible about the factors that may affect quality of life. However, the majority of quality of life research has focused on people with less severe dementia, and especially those who can provide self-ratings. Comparison of self- and proxy ratings has shown that proxy ratings are typically more negative than self-ratings [3–7]. Estimations of quality of life are subjective, and self-rating is the gold standard, but where dementia has progressed to the extent that verbal communication is very limited or completely absent, and self-rating is not possible, quality of life can only be assessed through direct observation or proxy rating [8]. In this situation it is important to be able to employ suitable measures and to be aware of factors that may affect or bias the proxy ratings made using these measures.

A recent systematic review identified 5 dementia-specific measures of quality of life developed for use with people who have severe dementia, but noted a lack of studies using these measures that can contribute information about their psychometric properties and relative merits [8]. One of the measures considered worthy of further investigation was the Quality of Life in Late-stage Dementia scale (QUALID) [9]. The QUALID is based on observable behaviours and contains 11 items asking respondents to rate the frequency with which they have observed these behaviours in the person with dementia over the previous week. In the original development study, the scale showed good inter-rater and test-retest reliability, and principal components analysis (PCA) identified a single factor. Studies have further examined the properties and applicability of the QUALID in Sweden [10], Spain [11], and Norway [12], supporting the reliability and validity of this measure, although with some variability in identified factor structure. Furthermore the QUALID appears sensitive to change resulting from both pharmacological and non-pharmacological interventions, and hence has the potential to serve as an outcome measure [13–15]. Additional information about this measure and its utility would therefore be valuable.

All studies involving the QUALID to date have been based on proxy ratings of resident quality of life made by members of care staff in residential and nursing home settings. However, the need to incorporate consideration of proxy ratings by family members when assessing quality of life in people with severe dementia has been noted [8]. A number of quality of life studies using other measures with people with dementia of varying degrees of severity in both community and residential settings have included both family and care staff ratings, but while many of these studies have examined which factors are associated with or predictive of care staff ratings, only a few have examined which factors are associated with, or predictive of, family member ratings. In most cases the focus has been on comparing proxy ratings with self-ratings by the person with dementia (e.g. [4,7]) rather than on differences between care staff and family member ratings. However, where proxy rating is the only means of assessing quality of life, the question of who provides the rating and what this implies may be important [16]. Ratings by family members and care staff are typically correlated (e.g. [4,6]), but they are far from identical [2], and can be differentially sensitive to effects of intervention [15]. It has been suggested that each different perspective on resident quality of life is ‘relatively independent and somewhat unique’ ([17] p.27) and that resident quality of life should be explored from multiple perspectives. In order to extend the available evidence, it would be helpful to examine family members’ proxy ratings alongside those of care staff.

In this study we explore the profiles of quality of life generated through proxy ratings by care staff and family members of people with very severe dementia using the QUALID, and aim to identify what factors influence these ratings for each group. In so doing we examine further the properties of the QUALID and its applicability in a severely-impaired United Kingdom (UK) sample. The following specific questions will be addressed:

1. What is the profile of family and care staff ratings of resident quality of life using the QUALID, and which variables are associated with, or predictive of, each of these two sets of quality of life ratings?
2. What are the psychometric properties of the QUALID scale for two groups of respondents, care staff and family carers?

## Method

### Design

This paper reports a cross-sectional examination of factors associated with family member and care staff ratings of the quality of life of people with severe dementia, and examines the psychometric properties of the QUALID measure. The analysis uses data from the AwareCare study [18], including data from the initial, measure development phase [19] and data from the baseline assessments conducted for the randomised controlled trial of the awareness-based intervention [15]. The relevant National Health Service and University ethics committees gave approval for each phase of the AwareCare study. As participants were unable to provide informed consent on their own behalf, in each case a relative was approached by the research team to act as a personal consultee as outlined in the provisions of the UK Mental Capacity Act (2005) [20], advising on whether or not the resident should be included in the study. In one case where no personal consultee was available, a nominated (non-kin) consultee was identified instead.

### Participants

**Figure 1:**
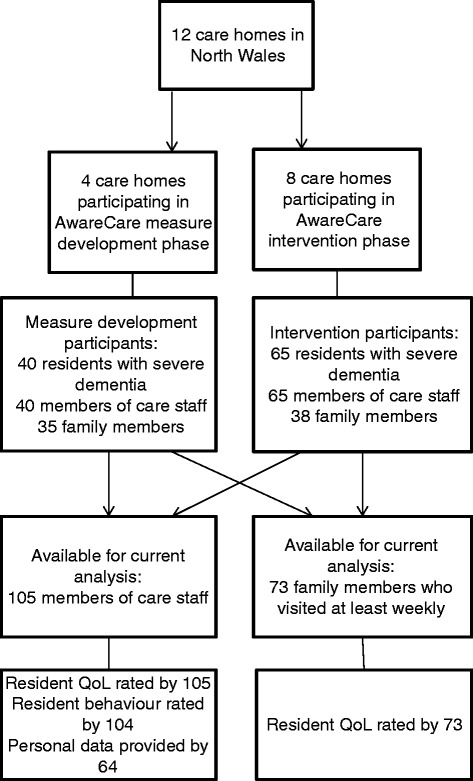
**Flowchart summarising data sources for this analysis.**

The participants were residents with severe dementia drawn from 12 care homes in North Wales, family members of these residents, and members of the care staff in the 12 care homes (see Figure 1). The study included 105 residents with severe dementia participating in AwareCare - 40 in the measure development phase and 65 in the randomised controlled trial (RCT). Seventy-three family members of these people with dementia provided proxy quality of life ratings. Also included were 105 members of care staff, of whom 40 rated resident quality of life and behaviour in the measure development phase, 64 contributed both proxy quality of life ratings, ratings of resident behaviour, and other personal data in the RCT phase, and 1 contributed quality of life ratings only in the RCT phase.

Inclusion criteria for residents were that they should meet criteria for Functional Assessment Staging (FAST) [21] stages 6 or 7, and should have no, or only very limited, verbal communication, indicated by an inability to clearly verbally communicate needs and wishes, with speech either very circumscribed and limited to single words or phrases or completely absent. Potential participants were identified, and family members notified, by care home managers. Inclusion criteria for care staff in the RCT phase were that the staff member should be a permanent employee, working 15 hours or more per week, who had been in post for at least two months, and should have good knowledge of the resident for whom proxy ratings were provided. Family carers were eligible to provide proxy quality of life ratings if they visited their relatives at least weekly; 76 family members met this criterion and 73 of these agreed to provide ratings. The 12 care homes were all privately owned; 11 offered both residential and nursing care and 1 offered only residential care. Eight homes specialised in dementia care while 4 offered care to older people with and without dementia.

### Measures

#### (a) Measures for the person with dementia

##### Residents’ personal details

The following information was collected for each resident: age, duration of stay in the care home, FAST stage, prescription of psychotropic and dementia medication, relationship of the resident to the family carer, and the frequency of visits by the family carer.

##### Quality of Life in Late-stage Dementia Scale (QUALID)

The QUALID [9] is an 11-item scale completed by a proxy with reference to the person’s quality of life in the preceding week. Perceived frequency of occurrence of the 11 behaviours or responses is rated on a 5-point scale. For 5 positively-stated items (smiles, enjoys eating, enjoys touching/being touched, enjoys interacting with others, appears calm and comfortable) 1 indicates the highest frequency and 5 the lowest, and for the remaining 6 negatively-stated items (appears sad, cries, facial expression of discomfort, appears physically uncomfortable, verbalisation suggests discomfort, is irritable and aggressive) 1 indicates the lowest frequency and 5 the highest. Thus, lower scores on this measure are indicative of better perceived quality of life. Independent proxy ratings of resident quality of life were made by a family member (where available and willing) and by a member of care staff.

##### Behavioural Assessment Scale of Later Life (BASOLL)

The BASOLL [22] is a reliable and valid measure of self-care ability, functioning and behavior. This measure, rated by care staff, incorporates sub-scales assessing mood (9 items), self-care (10 items), sensory abilities (2 items), memory and orientation (9 items), mobility (1 item) and challenging behaviour (5 items). Each item is rated on a 0 – 3 scale where higher score indicates greater severity of problems. Therefore, lower total scores for each subscale indicate fewer problems in that domain.

##### Guy’s Advanced Dementia Schedule (GADS)

The GADS [23], administered by a researcher, is a valid and reliable structured assessment of cognitive ability for people with severe dementia that involves measuring responses (reading, naming, using and taking) to familiar objects (such as a comb and a cup). Possible scores range from 0 – 40 with higher scores indicating greater cognitive ability. Up to three prompts may be given in relation to each item and in the present study three prompts were required in almost all cases.

##### Positive Response Scale (PRS)

The PRS [24] is an observational scale which focuses on the person’s affective response to the environment using 10 behavioural categories. Observations were conducted by a researcher, using a time-sampling schedule of one minute in every five over two 30-minute sessions, giving a total of 12 minutes of observation. This yielded a score representing the sum of the number of behaviours (out of the 10 possible categories) that occurred during each minute of the 12 minute observation period. This was divided by the total number of possible behaviours that could have been recorded (i.e. 10 behaviours × 12 minutes = 120) and the result was multiplied by 100 to give a percentage score. Higher percentage scores indicate greater frequency of observed behaviours.

##### *AwareCare*

The AwareCare observational measure [19] lists 10 events that either occur spontaneously in the environment (7 types, e.g. resident is touched, loud noise) or are introduced by the observer (3 types, e.g. resident is addressed by name). It has 14 response categories grouped into the following sub-categories: eyes (e.g. makes eye contact), face (e.g. smiles), head (e.g. nods/shakes head), arm (e.g. reaches), body (e.g. moves towards) and sounds (e.g. shouts or moans). For each event that occurs during the observation period, the observer records the resident’s response(s). The AwareCare responsiveness index (AwareCare RI) for each resident is the ratio of the number of responses made by the participant to the number of occurrences of events during the observation period. A higher RI indicates greater responsiveness. Ratings of resident awareness were made by a researcher in the measure development phase and by members of care staff in the RCT phase.

#### (b) Measures for care staff

##### Staff members’ personal details

The following information was collected for each staff member: age, gender, ethnicity (United Kingdom vs. European Community vs. other), first language (English/Welsh vs. other), duration of employment in care homes and duration of employment in the current home.

##### General Health Questionnaire (GHQ-12)

The GHQ [25] is a brief, well-validated, 12-item measure of psychological distress. Items are rated on a 0 – 3 scale and higher scores indicate higher levels of distress. Care staff used this scale to rate their general level of psychological distress.

##### Maslach Burnout Inventory (MBI)

The MBI [26] is a 25-item self-report questionnaire comprising subscales for emotional exhaustion (9 items), depersonalisation (5 items) and personal accomplishment (8 items) and three additional optional items reflecting involvement. Items are rated for both frequency and intensity, with higher scores indicating a greater sense of emotional exhaustion, depersonalisation or personal accomplishment. Care staff used this scale to rate aspects of their own well-being in relation to their work. Scores for the three subscales are reported here.

##### Approaches to Dementia Questionnaire (ADQ)

The ADQ [27] is a 19-item reliable and valid scale with two sub-scales assessing person-centred (11 items) and hopeful (8 items) attitudes to people with dementia. Higher scores indicate more positive attitudes towards people with dementia. Care staff completed this scale to provide an indication of their attitudes towards people with dementia.

### Procedure

For each resident, wherever possible, a family member was interviewed to provide background information about the resident and proxy ratings of quality of life (QUALID), and in each case a member of the care staff was interviewed separately to provide ratings of behaviour (BASOLL) and proxy ratings of quality of life (QUALID). Each member of the care staff gave ratings for one resident only, and in each case this was a resident who was well-known to the staff member. Each resident was then observed by a researcher for two 30-minute periods using the PRS to provide a profile of current well-being, and was assessed with the GADS to provide a profile of cognitive functioning. Care staff participating in the RCT phase also completed the GHQ-12, MBI and ADQ; the 40 residents in the initial, measure development stage did not have the staff personal information recorded.

Following these initial assessments, participants were observed using the AwareCare measure. In the measure development phase, these observations were conducted by the researchers during five 30-minute sessions with each of the 40 residents. In the RCT phase, following appropriate training, care staff in the four homes randomised to the intervention condition conducted the observations. Each staff member was asked to observe several residents according to a pre-planned schedule involving six 10-minute observations each week for six weeks, and observations were available for 32 residents. The mean number of observations obtained for each resident was 22 (s.d. 12.12, range 1 – 54). The mean number of observations conducted by each staff member across all assigned residents was 23 (s.d. 10, range 3 – 36), and the mean number of observations conducted by each staff member for individual assigned residents was 4.17 (s.d. 1.57, range 1 – 9). The AwareCare measure score was not available for the 33 residents in the four homes allocated to the control condition in the RCT.

### Data analysis

Quality of life ratings made by family members and care staff were compared using a paired-sample t-test. Kurtosis was within acceptable limits for the two sets of ratings, but skewness was slightly raised for family carers only (skewness = 1.203, se 0.281). Therefore a Wilcoxon test, the equivalent non-parametric test, was also applied; this gave an identical result to the paired-sample t-test, and hence the latter is reported below. Pearson’s r was calculated to determine the extent of correlation between the two sets of ratings. Analysis of variance was used to check for differences in ratings according to care home or resident gender.

The relationships of resident and staff measures with family carer and staff member ratings of quality of life were examined using correlation and regression analysis. Pearson’s r was calculated for continuous variables, and the point-biserial correlation for categorical variables. All regression analyses reported here used the stepwise method and were conducted with the default entry probability of p < 0.05 and the default removal probability of p > 0.10. Nominal and ordinal variables were dichotomised and coded 0/1 for inclusion in the correlation and regression analyses; the variable name given in the tables refers to the group coded ‘1’.

For family carer ratings, the regression analysis was initially run including all the resident personal and questionnaire variables, and was then re-run excluding the AwareCare and GADS measures to increase the sample size.

For care staff ratings, in view of the sample size, two separate regression analyses were conducted. The first used the resident personal information and questionnaire ratings as predictors, and the second used the care staff information and ratings as predictors. The significant predictor variables emerging from these two analyses were then combined in a further stepwise regression analysis to examine key predictors of care staff ratings of resident quality of life.

Exploratory factor analysis to examine the psychometric properties of the QUALID when rated by family carers and care staff was undertaken using Principal Components Analysis (PCA). In each case, item-scale reliability was examined using corrected item-total correlations, and Cronbach’s alpha was calculated prior to conducting the principal components analysis. Suitability of the data for PCA was determined with the Kaiser-Meyer-Olkin measure of sampling adequacy (0.702 for care staff and 0.686 for family carers), and Bartlett’s Test of Sphericity (approx. χ^2^_55_ 251.916, p < .001 for care staff and approx. χ^2^_55_ 223.912, p < .001 for family carers). The Kaiser-Meyer-Olkin statistic indicates the proportion of variance that may be caused by underlying factors; values above 0.5 suggest that the data may be amenable to factor analysis. Bartlett’s test of sphericity tests whether the correlation matrix is an identity matrix indicating that variables are unrelated and hence unsuitable for structure detection; significance levels < .05 indicate that a factor analysis may be appropriate. Therefore, although the family carer sample was relatively small, these indices gave no cause for concern regarding the suitability of the data for factor analysis. PCA was conducted using both the varimax with Kaiser normalization and the oblimin rotation methods. The two methods produced almost identical results, and the results from the varimax rotation method are reported here. Factors were initially selected based on an *a priori* criterion of eigenvalue > 1 and the final selection was determined following scrutiny of the scree plot and identification of inflection points. Structural validity of the identified factors was examined using Cronbach’s alpha (ά).

All analyses were conducted in IBM SPSS Statistics v20.

## Results

Descriptive details for the 105 residents and for the 64 members of care staff who contributed in the RCT phase are provided in Table 1. The majority of residents were female and most had FAST scores in stage 7. Prescription of antipsychotic medication was common. The family carers who completed the QUALID were more likely to be adult children than spouses; all visited the resident regularly, mostly once a week. Participating members of care staff were mostly female and from the UK, but 21 out of 64 (33%) were from outside the UK and 20 (31%) had a first language other than English or Welsh.

**Table 1:** **Descriptive profile of residents, family carers and care staff**

**Variable**	**Mean (s.d., range) or frequency**
**RESIDENTS** n = 105	
Age	Mean 81.47 years (s.d. 8.63, range 56 – 100)
Gender	72 (69%) female
	33 (31%) male
Ethnicity	104 (99%) white British
	1 (1%) white Irish
Diagnosis	40 (38%) dementia
	26 (25%) Alzheimer’s
	19 (18%) vascular dementia
	6 (6%) mixed Alzheimer’s and vascular
	3 (3%) fronto-temporal dementia/Pick’s
	1 (1%) CADASIL
	10 (9%) not stated
FAST stage	25 (24%) Stage 6 (2 6a, 2 6b, 4 6c, 2 6d, 15 6e)
	80 (76%) Stage 7 (27 7a, 28 7b, 14 7c, 7 7d, 4 7e)
Psychotropic medication prescribed	73 (70%) yes, 32 (30%) no
Antipsychotic	40 (38%) yes, 65 (62%) no
Benzodiazepine	32 (30%) yes, 73 (70%) no
Hypnotic	29 (28%) yes, 76 (72%) no
Antidepressant	33 (31%) yes, 72 (69%) no
Antiepileptic	9 (9%) yes, 96 (91%) no
Number of medication types prescribed	32 (30%) none
	25 (24%)1 type
	29 (28%) 2 types
	16 (15%) 3 types
	3 (3%) 4 types
Dementia medication prescribed	15 (14%) yes, 90 (86%) no
Length of stay in the home (months)	Mean 38.69 (s.d. 32.63; range 3 – 223)
**FAMILY CARERS** n = 105	
Relationship to resident	30 (29%) spouse
	58 (55%) child
	16 (15%) other relative
	1 (1%) none (resident had no relatives)
Frequency of visiting (data available for 73 carers who completed the QUALID)	41 (56%) 1 – 2 times a week
	15 (21%) 3 – 4 times a week
	4 (5%) 5 – 6 times a week
	8 (11%) daily
	5 (7%) ‘regularly’
**CARE STAFF** n = 64	
Age	Mean 38.16 years (s.d. 9.87, range 21 – 57)
Gender	51 (80%) female
	13 (20%) male
Ethnicity	43 (67%) UK
	6 (10%) EC
	15 (23%) other
First language	44 (69%) English/Welsh
	20 (31%) other
Length of time working in care sector (years)	Mean 8.23 (s.d. 7.65, range 0.17 – 30)
Length of time working in this home (years)	Mean 4.70 (s.d. 4.86, range 0.17 – 21)
Qualifications in care provision	12 (19%) no qualifications
	3 (5%) NVQ Level 1
	19 (31%) NVQ level 2
	21 (33%) NVQ Level 3
	9 (14%) nursing qualification

*Note.* FAST: Functional Assessment Staging; CADASIL: Cerebral Autosomal-Dominant Arteriopathy with Subcortical Infarcts and Leukoencephalopathy; QUALID: Quality of Life in Late-stage Dementia scale; UK: United Kingdom; EC: European Community; NVQ: National Vocational Qualification.

The profile of quality of life ratings by family carers and care staff will first be described, and correlates and predictors of these ratings examined. The psychometric properties of the QUALID in the AwareCare study will then be outlined. Details of scores on all measures are shown in Table 2.

**Table 2:** **Scores on resident and care staff measures**

**Measure**	**N**	**Mean**	**S.D.**	**Range**	**Worst-best**
**Residents**					
QUALID rated by family member	73	21.66	6.71	11 - 40	55 - 11
QUALID rated by member of care staff	105	21.96	6.21	12 - 39	55 - 11
BASOLL self-care	105	23.85	5.34	2 – 30	30 - 0
BASOLL memory and orientation	105	10.78	3.73	0 – 19	27 - 0
BASOLL challenging behaviour	105	3.55	3.26	0 – 15	15 - 0
BASOLL mood	105	2.35	2.62	0 – 12	27 - 0
BASOLL sensory abilities	105	1.21	1.04	0 – 6	6 - 0
BASOLL mobility	105	1.18	1.02	0 – 3	3 - 0
GADS	96	9.98	6.37	0 – 23	0 - 40
PRS (%)	105	38.25	12.45	10 – 66.67	0 - 100
AwareCare RI	72	2.95	1.11	.89 – 5.54	Higher better
**Care staff**					
GHQ-12	64	8.30	4.25	0 - 20	36 - 0
MBI emotional exhaustion	64	13.14	10.77	0 - 36	54 - 0
MBI depersonalisation	64	2.50	3.21	0 – 14	30 - 0
MBI personal accomplishment	64	37.64	9.30	2 - 48	0 - 48
ADQ hope	64	27.67	4.88	15 – 37	8 - 40
ADQ person-centredness	64	48.69	4.37	33 - 55	11 - 55

*Note.* Worst-best: range of scores from the poorest possible to the best possible score (where applicable); QUALID: Quality of Life in Late-stage Dementia Scale; BASOLL: Behavioural Assessment Scale of Later Life; GADS: Guy’s Advanced Dementia Schedule; PRS: Positive Response Scale; AwareCare RI: AwareCare Responsiveness Index; GHQ: General Health Questionnaire; MBI: Maslach Burnout Inventory; ADQ: Approaches to Dementia Questionnaire.

### Profile of quality of life scores

Mean scores for the two sets of QUALID ratings were very similar (lower scores on the QUALID indicate better quality of life). For those residents where quality of life was rated by both a family carer and a member of the care staff (n = 73), mean ratings were slightly higher (less positive) for care staff than for family carers (22.59 ± 6.56 vs. 21.66 ± 6.71), but a paired-samples t-test indicated no significant difference in the means (t_72_ = −1.106, p = .273), and the two sets of scores were moderately and significantly correlated (r = .412, p = .000). Neither family member nor care staff ratings differed by care home (F_11,61_ = .938, p > .05 for family carer ratings; F_11,93_ = 1.732, p > .05 for care staff ratings) or according to the gender of the resident (F_1,71_ = .011, p > .05 for family carer ratings; F_1,103_ = .444, p > .05 for care staff ratings).

### Factors associated with family carer ratings

Family carer QUALID ratings were available for 73 residents. Table 3 shows the correlations between these ratings and resident personal details and questionnaire scores. The only significant association was with the resident variable of prescription of antipsychotic medication; residents who were prescribed antipsychotic medication were regarded as having poorer quality of life. The regression analysis was initially run including all 22 of the resident personal and questionnaire variables listed in Table 3, and antipsychotic medication emerged as the only predictor variable. However, the sample size was only 49 due to missing values for the AwareCare or GADS measures. As neither variable was included in the model, the regression analysis was re-run excluding these two variables to increase the sample size, but again only antipsychotic medication was identified as a significant predictor. More positive family carer QUALID ratings were significantly predicted by non-use of antipsychotic medication (coefficient = 3.744, SD = 1.623, beta = 0.268, t(70) = 2.306, p = .024, adjusted R^2^ = .058).

**Table 3:** **Correlates of family member QUALID ratings (n = 73)**

**Resident personal information**			**Resident questionnaire ratings**		
	QUALID family carer score	p		QUALID family carer score	p
Age	.008	.945	BASOLL self-care score	.194	.100
Male	.012	.917	BASOLL memory and orientation score	-.012	.923
Time in care home (months)	-.144	.225	BASOLL challenging behaviour score	.116	.329
FAST stage 7	.146	.217	BASOLL mood score	.114	.335
Antipsychotic medication	.252^*^	.032	BASOLL sensory abilities score	.077	.515
Benzodiazepine medication	.062	.601	BASOLL mobility score	.083	.484
Hypnotic medication (other than benzodiazepine)	-.142	.230	GADS score (n = 67)	-.098	.428
Antidepressant medication	.053	.655	Positive Response Scale score	-.111	.351
Medication for seizures or bipolar disorder	.090	.447	AwareCare Responsiveness Index score (n = 53)	-.052	.713
Number of psychotropic medications	.124	.294			
Dementia medication	.184	.118			
Not spouse (n = 72)	-.044	.714			
frequent visits (n = 72)	.096	.424			

Note. *denotes significant at the p < .05 level.

### Factors associated with care staff ratings

Table 4 shows the correlations of care staff QUALID ratings (n = 105) with resident personal information and resident questionnaire ratings. Care staff QUALID ratings were significantly associated with the resident variables of FAST stage, prescription of benzodiazepine medication, and number of types of psychotropic medication prescribed. Residents who were more impaired, were prescribed benzodiazepines or were prescribed more types of psychotropic medication were regarded as having poorer quality of life. With regard to the questionnaire measures, there were significant associations with the AwareCare RI score and with the BASOLL self-care, mood and challenging behaviour scores. Greater responsiveness to stimuli as shown by the AwareCare RI, BASOLL scores indicative of fewer difficulties in self-care, mood and behaviour, and the non-use of benzodiazepines were all associated with better QUALID ratings. Table 4 also shows the correlations between care staff variables and QUALID scores for the 64 care staff participating in the AwareCare RCT phase. The only variables significantly associated with staff ratings of resident quality of life were the staff member’s ethnicity and first language. This suggests that staff identifying as British and having English or Welsh as their first language tended to rate resident quality of life more positively than staff from overseas.

**Table 4:** **Correlates of care staff QUALID ratings (n = 105)**

**Resident personal information**			**Resident questionnaire ratings**			**Care staff personal information and ratings (n = 64)**		
	QUALID staff member score	*p*		QUALID staff member score	*p*		QUALID staff member score	*p*
Age	-.148	.132	BASOLL self-care score	.256**	.008	Care staff member’s age	-.113	.375
Male	-.065	.507	BASOLL memory and orientation score	.119	.226	Staff male	.230	.067
Time in care home (months)	-.080	.415	BASOLL challenging behaviour score	.362**	<.001	Staff ethnicity	.275*	.028
FAST stage	.208*	.033	BASOLL mood score	.356**	<.001	Foreign language	.305*	.014
Antipsychotic medication	.170	.083	BASOLL sensory abilities score	.109	.269	Care staff member’s qualifications in care	.031	.805
Benzodiazepine medication	.315**	.001	BASOLL mobility score	.138	.160	Time working in care homes (years)	-.068	.593
Hypnotic medication (other than benzodiazepine)	-.079	.424	GADS score (n = 96)	-.146	.156	Time working in this care home (years)	-.124	.329
Antidepressant medication	.027	.781	Positive Response Scale score	-.038	.698	GHQ total	-.019	.880
Medication for seizures or bipolar disorder	.079	.424	AwareCare Responsiveness Index score (n = 72)	-.331**	.005	MBI emotional exhaustion sub-scale	-.233	.064
Number of psychotropic medications	.198*	.043				MBI depersonalisation sub-scale	-.036	.779
Dementia medication	.086	.382				MBI personal accomplishment sub-scale	-.039	.760
						ADQ hope sub-scale	.007	.957
						ADQ person-centredness sub-scale	.226	.072

Note. *denotes significant at the p < .05 level; **denotes significant at the p < .01 level.

Only 30 residents had full data for all 33 predictor variables shown in Table 4, so it was not advisable to run a regression analysis using all variables, and two separate analyses were undertaken. Firstly we used the resident personal information and questionnaire ratings as predictors (n = 67), and secondly we used the care staff information and ratings as predictors (n = 64). Using the 20 resident variables, the regression analysis for predicting care staff QUALID rating included AwareCare RI, prescription of benzodiazepine medication, and BASOLL mood score. Table 5 shows the coefficients and adjusted R^2^ values. Using the 13 care staff variables the regression analysis included the care staff member’s first language, the MBI emotional exhaustion sub-scale score, and the GHQ score. This suggests that better QUALID ratings were associated with more emotional exhaustion, greater individual psychological well-being, and also with having English or Welsh as the first language, in care staff. Table 6 shows the coefficients and adjusted R^2^ values.

**Table 5:** **Variables chosen by stepwise regression of resident factors on care staff QUALID ratings (N = 67)**

	**Coefficient**	***SD***	***Beta***	***t***	***p***
AwareCare responsiveness index score	−2.056	0.561	−0.391	−3.662	.001
Benzodiazepine medication	3.524	1.318	0.305	2.674	.010
BASOLL mood score	0.566	0.279	0.232	2.027	.047
R^2^ = .300, adjusted R^2^ = .267

**Table 6:** **Variables chosen by stepwise regression of care staff factors on care staff QUALID ratings (N = 64)**

	**Coefficient**	***SD***	***Beta***	***t***	***p***
First language other than English/Welsh	5.86	1.671	0.435	3.508	.001
MBI emotional exhaustion sub-scale	−0.237	0.077	−0.406	3.084	.003
GHQ total	0.416	0.202	0.281	2.057	.044
R^2^ = .402, adjusted R^2^ = .361					

The six significant predictor variables identified in Tables 5 and 6 were combined in a further stepwise regression analysis to examine key predictors of care staff ratings of resident quality of life. The sample size for this analysis was 32. The predictor variables chosen were BASOLL mood score (p = .003) and AwareCare RI (p = .003), with an adjusted R^2^ value of .361. Therefore, greater responsiveness and more positive mood were predictive of more positive quality of life ratings.

### Psychometric properties of the QUALID

The psychometric properties of the QUALID were examined separately for the two groups of respondents, care staff and family carers. Details of item-scale reliability and factor structure are shown in Table 7.

**Table 7:** **Factor structure of the QUALID for the care staff (n = 105) and family carer (n = 73) samples, and corrected item-scale correlations**

**Scale item**	**Care staff**		**Care staff**	**Family carers**		**Family carers**
	Factor 1	Factor 2		Factor 1	Factor 2	
	Discomfort and distress	Sociability	Item-scale correlations	Discomfort and distress	Sociability	Item-scale correlations
1. Smiles		.713	.118	.230		.201
2. Appears sad	.702		.492	.676		.465
3. Cries	.518		.280	.365		.237
4. Facial expression of discomfort	.562		.341	.656		.465
5. Appears physically uncomfortable	.533		.350	.653		.407
6. Verbalisation suggests discomfort	.757		.532	.846		.702
7. Is irritable and aggressive	.651		.427	.567		.371
8. Enjoys eating	.169		.073	.170		.048
9. Enjoys touching/being touched		.825	.146		.819	.381
10. Enjoys interacting with others		.827	.186		.843	.128
11. Appears calm and comfortable	.776		.538	.736		.496

For responses by family carers, corrected item-total correlations ranged from .048 for item 8, ‘enjoys eating’, to .702 for item 6, ‘verbalisation suggests discomfort’. In total, 9 items had correlations > .2 and 4 had correlations > .4; apart from ‘enjoys eating’, the exception was item 10, ‘enjoys interacting with others’. Cronbach’s alpha for the whole scale was .701. Removing item 8, ‘enjoys eating’, increased this only slightly to .713. Three factors with eigenvalues > 1 were identified and their suitability was confirmed by examination of the scree plot. These were labelled as follows: Factor 1 – discomfort and distress (8 items; ά = 0.746); Factor 2 – emotional state (4 items; ά = 0.496); Factor 3 – sociability (2 items; ά = 0.749). In view of the relatively low Cronbach’s alpha for Factor 2, the analysis was repeated stipulating a two-factor solution. The two factors derived were as follows: Factor 1 – discomfort and distress (9 items; ά = 0.71) and Factor 2 – sociability (2 items; ά = 0.749). Table 7 shows which items primarily loaded onto each of these factors.

For responses by care staff, corrected item-scale correlations ranged from .073 for item 8, ‘enjoys eating’ to .538 for item 11, ‘appears calm and comfortable’. In total, 7 items had correlations > .2 and 4 items > .4. Apart from ‘enjoys eating’, the remaining items correlating < .2 were item 1, ‘smiles’, item 9, ‘enjoys touching/being touched’, and item 10, ‘enjoys interacting with others’. Cronbach’s alpha for the whole scale was .67. Removing item 8, ‘enjoys eating’, increased this only slightly to .678. Four factors with eigenvalues > 1 were initially identified. However, factor 3 had only two items that primarily loaded onto it while factor 4 had only one, item 8, ‘enjoys eating’. Examination of the scree plot indicated that these two factors had eigenvalues only slightly > 1 and that the major inflection point occurred between factors 2 and 3. Therefore the analysis was repeated stipulating a three-factor solution. In this solution, factor 3 again contained only item 8, ‘enjoys eating’, and so the analysis was repeated stipulating a two-factor solution. This yielded two factors which were labelled as follows: Factor 1 – discomfort and distress (8 items; ά = 0.746), and Factor 2 – sociability (3 items; ά = 0.694). Table 7 shows which items primarily loaded onto each of these factors. This differed from the two-factor solution for family carers only in that for family carers item 1, ‘smiles’, loaded onto Factor 1 (discomfort and distress) rather than Factor 2 (sociability).

## Discussion

This study is one of few to focus on people with very severe dementia who have no, or only very limited, verbal communication, with the aim of understanding more about care staff and family carer perceptions of quality of life in this group and what factors influence these perceptions. The study was the first to examine family carer proxy ratings using the QUALID and the first to examine the role of awareness/responsiveness and staff ethnicity in influencing proxy ratings. This study also provided an opportunity to examine the psychometric properties of the QUALID scale when rated by family carers and by care staff, and its applicability with a UK sample of severely impaired residents.

The overall profile of quality of life was broadly consistent with that reported in other studies. Mean scores on the QUALID, rated by both family members and care staff, were slightly more positive in this sample than those reported in previous studies [9–12]. It is not clear why this was the case, as all studies sampled from long-term dementia care settings, although one study also sampled from psychiatric hospital wards [12]. However, the samples in these previous QUALID studies were less impaired than in the present study. Despite characterising samples as having severe dementia, MMSE score ranges where quoted were wide (0 – 25 [10] and 0 – 30 [11]). In the present study, our inclusion criteria were such that participants would not have been expected to complete any items on the MMSE, and hence this measure was not used; the majority of participants met criteria for FAST stage 7. There is no consensus on a definition of ‘severe’ dementia [28], and clearly there is considerable variability in sample characteristics among studies purporting to investigate aspects of severe dementia. This limits the extent to which meaningful comparisons can be made. In many cases it seems that no cut-off is applied to represent the upper limit of ability in ‘severe’ dementia, which complicates the picture even further. Even if a cut-off score on the MMSE were to be applied, the differences between someone who scores 10 and is fully mobile and able to comment on his/her own quality of life and someone who is in FAST stage 7, unable to score on the MMSE, immobile and no longer communicating verbally are considerable. There is a need for more precise characterisation of samples and for greater homogeneity of samples in research studies in this field.

In our study, the only variable contributing to prediction of family member ratings was prescription of antipsychotic medication, accounting for 5.8% of variance. Few studies have examined predictors of family member ratings; none of these used the QUALID, and again the samples have tended to be less impaired than in the present study. One study in residential settings identified cognition, health problems and behavioural symptoms of the person with dementia, and use of restraint, as predictive of family member ratings [4]. Another identified resident functional ability, carer contribution to nursing home costs and use of feeding tubes as relevant, accounting for 25.1% of variance, but found no effect of carer stress or emotional well-being [7]. In a sample recruited initially from general hospitals, there was no association between carer stress or psychological distress and proxy ratings of quality of life [5], although these factors were associated with the person’s self-ratings of quality of life; factors influencing carer ratings were the person’s functional ability and dementia severity. One study with a community sample identified predictors of proxy quality of life ratings by family members as cognition, functional ability, neuropsychiatric symptoms, depression and prescription of antipsychotic medication together with carer burden and relationship to the person with dementia, accounting for 59.8% of variance [29]. Family members of community-dwelling people with dementia might be expected to be more directly involved in care and hence more aware of functioning and symptoms, but family carers who provided proxy ratings in our study visited their relatives at least weekly, and therefore had regular opportunities to observe residents’ functioning and well-being. A possible explanation for the limited proportion of variance accounted for in the present study could be the severity of impairment in the present sample, as different factors may come into play in very severe dementia. In the AwareCare trial [15], family member ratings of quality of life were sensitive to change whereas care staff ratings were not, which is consistent with the lack of overlap in predictive variables and supports the view that the two sets of ratings are independent and influenced by different factors [17].

The key predictors of care staff ratings in this study were resident mood, awareness/responsiveness (indicated by the AwareCare RI), and prescription of benzodiazepine medication, together accounting for 26.7% of variance. Our findings on resident factors are broadly consistent with those from other studies in long-term care settings, although the samples in many of these studies were less impaired than the present sample. Most studies using the QUALID provide only correlational analyses, indicating associations with mood [9,10], neuropsychiatric symptoms and behaviour [9–11], cognition [10,11] and pain [11]; one study reporting a regression analysis found that key predictors were mood and functional ability [12]. Factors identified in previous studies as predictors of staff ratings on other quality of life measures are resident mood, cognition, functional ability or dependency, neuropsychiatric symptoms and behavioural difficulties [1,4,30–33]. In some studies psychotropic drug use, physical health problems and falls [4] also contribute; in our study it was specifically use of benzodiazepines that was associated with care staff quality of life ratings. Inclusion of the AwareCare measure provides novel evidence indicating that resident responsiveness to stimuli and interactions is strongly linked to care staff evaluations of quality of life. While the QUALID involves assessment of observable behaviours and responses, estimates of frequency of occurrence over the past week will necessarily be somewhat subjective, and in addition items require some judgements, for example regarding whether the resident shows ‘enjoyment’. AwareCare is based on direct behavioural observation and on clearly-defined bodily responses, and the association with quality of life ratings could be considered to support the construct validity of the QUALID. It is understandable that staff might hold more positive views of residents who are more likely to respond to, and interact with, them. It has been suggested previously that where staff perceive residents as having the capacity for relationships and activities, they will also ascribe a good quality of life [32]. Nevertheless it is important to exercise caution in equating awareness/responsiveness with quality of life, as we should not necessarily assume that non-responsiveness indicates negative internal states.

The effects of care staff variables were also examined. When considering care staff variables alone, extent of emotional exhaustion, lower psychological distress and language were individually significant predictors, and together these accounted for 36.1% of variance. Our tentative finding, albeit based on a smaller sample size (n = 32), that staff factors are weaker predictors than resident factors is consistent with the few previous studies that have included an examination of care staff factors [4,7,32]. Staff distress at neuropsychiatric symptoms [4] and nursing assistant characteristics including attitudes to dementia [32] accounted for only a small proportion of variance in overall regression models. In one study emotional exhaustion, job satisfaction, training and experience were not related to staff ratings of quality of life, although staff shift pattern (permanent vs. rotating) and type of home (private vs. public) contributed alongside resident functional ability, cognition and mood in a regression model accounting for 41.3% of variance [7]. The impact of staff ethnicity has not previously been examined and our data contribute a novel perspective indicating that where staff member and resident share the same ethnic background, staff rate quality of life more positively. Possible explanations for this might be that ratings are influenced by cultural beliefs and assumptions about care for older people and people with dementia, or that communication is more effective where staff are native speakers of the resident’s language.

With regard to the properties of the QUALID, Cronbach’s alpha was slightly lower in our study than in the previous QUALID studies [9–11]. Item-scale reliability indicated that item 8, ‘enjoys eating’, had a very weak correlation with the overall total score in both groups of respondents. This item did not emerge as causing concern in other studies reporting item-scale reliability data [9–11]. Items causing concern in earlier studies were item 3, ‘cries’ [9,10] and item 7, ‘appears irritable and aggressive’ [11]. Items causing more concern in the present study were item 10, ‘enjoys interacting with others’, which was weakly correlated for both care staff and family members, and item 9, ‘enjoys touching/being touched’ which was weakly correlated in the care staff ratings. Adopting a criterion of taking only correlations of 0.4 or above as supporting internal consistency [11], from available data one study had 4 items reaching this criterion [10] and one had 5 [11]. Our results were consistent with this. These findings indicate that there is a small degree of variability in the internal consistency of the measure across studies; one possible explanation is that this may be attributable to cross-national differences, but it is also important to note that the samples in previous studies tended to be less severely impaired than the present sample. Nevertheless, our data supports the applicability of the QUALID.

Factor analysis produced similar results for family members and care staff. Previous studies of factor structure in care staff responses have been inconsistent. The original development study [9] and the subsequent evaluation of the Swedish version [10] suggested a single factor. However, subsequent studies reported a two-factor [12] or a three-factor [11] solution. Reviewing the factor structures identified in these two studies and in the two groups of respondents in the present study, it appears that two main components can be distinguished. Seven items (2, 3, 4, 5, 6, 7, 11) seem to share common elements even though the factor labels may differ slightly, as these items load onto factors labelled ‘discomfort’, ‘discomfort or distress’, or ‘negative mood’, encapsulating the presence or absence of negative physical and emotional states. Three items (1, 9, 10) load onto factors labelled ‘social interaction’, ‘sociability’, or ‘comfort’, encapsulating the presence or absence of social communication, responsiveness and positive emotional states. Item 8, ‘enjoys eating’, shows most variability and does not fit reliably into either of these two groupings. Residents with severe dementia often lack appetite, may need help with eating, and are likely to be fed by care staff, and hence the question may have limited suitability for this group. In any future revision of the scale it may be useful to consider omitting or revising this item, as well as identifying ways of improving upon any other items found to have low internal consistency across studies using the QUALID.

Several limitations resulting from study design must be taken into consideration. Family carers were asked to provide ratings of resident quality of life only, and we were not able to collect information about factors pertaining to family carers themselves that may have been associated with their proxy ratings of quality of life. The ability to respond accurately to items on the QUALID depends on frequent observation and hence may have been somewhat challenging for the majority of family members who visited once or twice a week. Sample size was restricted for family carer QUALID ratings; as the scale asks about observations during the past week, we were only able to obtain ratings from family members who visited regularly. Sample size was also restricted for care staff variables as the relevant measures were used only in the intervention phase of the study, and for the residents’ AwareCare RI score as no observations were available for participants residing in care homes that took part in the intervention trial but were allocated to the control condition. However, although our sample size was relatively small to undertake regression analyses, by limiting the number of predictor variables in each model, post hoc power calculations suggested that we did retain sufficient power to detect medium to large effect sizes. The residents and care staff were drawn from 12 residential homes, but with an average of only 8.75 residents and carers from each home it was not feasible to examine whether facility-level factors had any effect on ratings, as has been suggested [7]. Nevertheless, despite these limitations, this study provides new evidence about factors affecting care staff perceptions of resident quality of life and indicates directions for further research, particularly with regard to factors related to family carer ratings. While the practicalities of obtaining ratings from residents’ family members can be challenging, it is important to include this distinct perspective alongside that of care staff in studies examining the quality of life of severely-impaired residents with dementia who are unable to communicate verbally regarding their own perceptions.

## Conclusions

The findings of this study offer novel evidence about predictors of care staff proxy ratings of quality of life, identifying resident mood and awareness/responsiveness as key predictors with a more limited contribution from staff emotional exhaustion and ethnicity. The findings further demonstrate that commonly-assessed resident variables explain little of the variability in family members’ proxy ratings of quality of life and point to a need to think more broadly about what might be salient for family members in this situation. This study contributes to the growing body of evidence about the suitability of the QUALID as a proxy measure of quality of life in severe dementia, and supports the applicability of this measure, which captures both negative and positive elements of observed experience. This study is one of very few to focus on a clearly-described group of residents with very severe dementia, and demonstrates a need for studies evaluating quality of life to identify more homogeneous samples. It is particularly important for research to pay careful attention to this group of people with very severe dementia who cannot speak for themselves and express their views about their own quality of life, so that appropriate ways of promoting quality of life can be identified and implemented.

## Abbreviations

ADQ: Approaches to Dementia Questionnaire
AwareCare RI: AwareCare Responsiveness Index
BASOLL: Behavioural Assessment Scale of Later Life
FAST: Functional Assessment Staging
GADS: Guy’s Advanced Dementia Schedule
GHQ: General Health Questionnaire
MBI: Maslach Burnout Inventory
PCA: Principal components analysis
PRS: Positive Response Scale
RCT: Randomised Controlled Trial
QUALID: Quality of Life in Late-stage Dementia Scale

## References

[1] Cordner Z, Blass DM, Rabins PV, Black BS (2010). Quality of life in nursing home residents with advanced dementia. J Am Geriatr Soc.

[2] Kane RA, Kling KC, Bershadsky B, Kane RL, Giles K, Degenholtz HB, Liu J, Cutler LJ (2003). Quality of life measures for nursing home residents. J Gerontol A Biol Sci Med Sci.

[3] Hoe J, Katona C, Roch B, Livingston G (2005). Use of the QOL-AD for measuring quality of life in people with severe dementia—the LASER-AD study. Age Ageing.

[4] Beer C, Flicker L, Horner B, Bretland N, Scherer S, Lautenschlager NT, Schaper F, Almeida OP (2010). Factors associated with self and informant ratings of the quality of life of people with dementia living in care facilities: a cross sectional study. PLoS One.

[5] Sheehan BD, Lall R, Stinton C, Mitchell K, Gage H, Holland C, Katz J (2012). Patient and proxy measurement of quality of life among general hospital in-patients with dementia. Aging Ment Health.

[6] Moyle W, Gracia N, Murfield JE, Griffiths SG, Venturato L (2012). Assessing quality of life of older people with dementia in long-term care: a comparison of two self-report measures. J Clin Nurs.

[7] Crespo M, Hornillos C, de Quirós MB (2013). Factors associated with quality of life in dementia patients in long-term care. Int Psychogeriatr.

[8] Moyle W, Murfield JE (2013). Health-related quality of life in older people with severe dementia: challenges for measurement and management. Expert Rev Pharmacoecon Outcomes Res.

[9] Weiner MF, Martin-Cook K, Svetlik DA, Saine K, Foster B, Fontaine CS (2000). The Quality of Life in Late-Stage Dementia (QUALID) scale. J Am Med Dir Assoc.

[10] Falk H, Persson LO, Wijk H (2007). A psychometric evaluation of a Swedish version of the Quality of Life in Late-Stage Dementia (QUALID) scale. Int Psychogeriatr.

[11] Garre-Olmo J, López-Pousa S, Turon-Estrada A, Juvinyà D, Ballester D, Vilalta-Franch J (2012). Environmental determinants of quality of life in nursing home residents with severe dementia. J Am Geriatr Soc.

[12] Barca ML, Engedal K, Laks J, Selbæk G (2011). Quality of life among elderly patients with dementia in institutions. Dement Geriatr Cogn Disord.

[13] Martin-Cook K, Hynan LS, Rice-Koch K, Svetlik DA, Weiner MF (2005). Responsiveness of the Quality of Life in Late-Stage Dementia Scale to psychotropic drug treatment in late-stage dementia. Dement Geriatr Cogn Disord.

[14] Schoelzel-Dorenbos CJM, Ettema TP, Bos J, Boelens-van der Knoop E, Gerritsen DL, Hoogeveen F, de Lange J, Meihuizen L (2007). Evaluating the outcome of interventions on quality of life in dementia: selection of the appropriate scale. J Geriatr Psychiatry.

[15] Clare L, Whitaker R, Woods RT, Quinn C, Jelley H, Hoare Z, Woods J, Downs M, Wilson BA (2013). AwareCare: a pilot randomized controlled trial of an awareness-based staff training intervention to improve quality of life for residents with severe dementia in long-term care settings. Int Psychogeriatr.

[16] Gräske J, Fischer T, Kuhlmey A, Wolf-Ostermann K (2012). Quality of life in dementia care – differences in quality of life measurements performed by residents with dementia and by nursing staff. Aging Ment Health.

[17] Edelman P, Fulton BR, Kuhn D, Chang CH (2005). A comparison of three methods of measuring dementia-specific quality of life: perspectives of residents, staff, and observers. Gerontologist.

[18] Clare L, Woods RT, Whitaker R, Wilson BA, Downs M (2010). Development of an awareness-based intervention to enhance quality of life in severe dementia: trial platform. Trials.

[19] Clare L, Whitaker R, Quinn C, Jelley H, Hoare Z, Woods RT, Downs M, Wilson B (2012). AwareCare: Development and validation of an observational measure of awareness in people with severe dementia. Neuropsychol Rehabil.

[20] Department of Constitutional Affairs (2007). The Mental Capacity Act, 2005. Code of practice.

[21] Reisberg B (1988). Functional assessment staging (FAST). Psychopharmacol Bull.

[22] Brooker DJR, Sturmey P, Gatherer AJH, Summerbell C (1993). The Behavioural Assessment Scale of Later Life (BASOLL): a description, factor analysis, scale development, validity and reliability data for a new scale for older adults. Int J Geriatr Psych.

[23] Ward T, Dawe B, Procter A, Murphy E, Weinman J (1993). Assessment in severe dementia: the Guy’s Advanced Dementia Schedule. Age Ageing.

[24] Perrin T (1997). The Positive Response Schedule for severe dementia. Aging Ment Health.

[25] Goldberg D (1992). General Health Questionnaire (GHQ-12).

[26] Maslach C, Jackson SE, Leiter MP (1996). Maslach Burnout Inventory.

[27] Lintern R, Woods RT, Phair L (2000). Training is not enough to change care practice. J Dementia Care.

[28] Koopmans RTCM, van der Molen M, Raats M, Ettema TP (2009). Neuropsychiatric symptoms and quality of life in patients in the final phase of dementia. Int J Geriatr Psychiatry.

[29] Mougias AA, Politis A, Lyketsos CG, Mavreas VG (2011). Quality of life in dementia patients in Athens, Greece: predictive factors and the role of caregiver-related factors. Int Psychogeriatr.

[30] González-Salvador T, Lyketsos CG, Baker A, Hovanec L, Roques C, Brandt J, Steele C (2000). Quality of life in dementia patients in long-term care. Int J Geriatr Psychiatry.

[31] Yamamoto-Mitani N, Abe T, Okita Y, Hayashi K, Sugishita C, Kamata K (2004). The impact of subject/respondent characteristics on a proxy-rated quality of life instrument for the Japanese elderly with dementia. Qual Life Res.

[32] Winzelberg GS, Williams CS, Preisser JS, Zimmerman S, Sloane PD (2005). Factors associated with nursing assistant quality-of-life ratings for residents with dementia in long-term care facilities. Gerontologist.

[33] Hoe J, Hancock G, Livingston G, Orrell M (2006). Quality of life of people with dementia in residential care homes. Br J Psychiatry.

